# The determinants of international students studying in China: An empirical research based on the “Belt and Road” perspective

**DOI:** 10.1371/journal.pone.0329787

**Published:** 2025-08-26

**Authors:** Tingsong Li, Xiaohua Zong, Wei Zhang

**Affiliations:** 1 School of Education, Shanghai Jiao Tong University, Shanghai, China; 2 Institute of Education, Nanjing University, Nanjing, China; 3 School of Education, University of Leicester, Leicester, United Kingdom; Chengdu Normal University, CHINA

## Abstract

This paper explores the factors influencing international students’ decisions to study in China, based on data from 2003 to 2018. Using a two-way fixed effect model and a comprehensive theoretical framework that integrates push-pull theory, human capital theory, and proximity theory, the study investigates the roles of economic, educational, national stability, and sociocultural determinants. The findings reveal that economic factors, particularly the trade relations between the home country and China, play a significant role in attracting international students. National stability, the number of Confucius Institutes, and language similarity also emerge as key influences. Moreover, educational factors such as Chinese university rankings and scholarships are crucial in drawing students. The study further shows that international students from B&R countries are more sensitive to economic and sociocultural factors than those from non-B&R countries. Postgraduate students are more focused on China’s economic development and educational strength, while undergraduates prioritize national stability and sociocultural aspects. These findings offer insights for policymakers and higher education institutions, providing strategic recommendations to effectively attract and support international students in a rapidly changing global landscape.

## Introduction

Since the early 21st century, the globalization of higher education has significantly accelerated due to the mobility of international students across various countries [1]. Evidence suggests that studying abroad enhances employability and contributes positively to the career development of international students. Consequently, an increasing number of students are opting to study abroad in pursuit of high-quality higher education [2].

**Fig 1:**
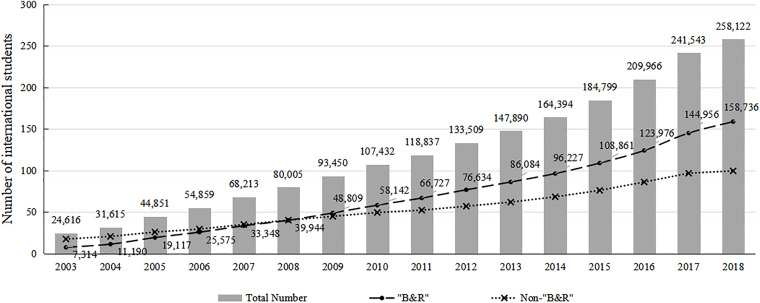
Number of international students in China by year. *Source*: Ministry of Education of China. (2003-2018).

From 2003 to 2009, the number of international students increased from 2.65 million to 3.54 million, and by 2022, it had grown to 6.86 million [3]. As the scale of international student mobility continues to expand, countries that accept most international students are also transforming. In 2009, the top three countries in terms of receiving international students were the United States, the United Kingdom, and Australia. However, to 2019, China has replaced Australia as the third largest destination for international students after the US and the UK [4]. In 2003, China had only 24,616 degree-seeking international students. As of 2018, the number has increased to 258,122, achieving an average annual growth rate of 16.97%. As shown in Fig 1, in 2003, 7,314 international students from B&R countries came to China to study. By 2018, the number had increased to 158,736, achieving an average annual growth rate of 23.70%, higher than the overall growth rate of international students [5]. Meanwhile, in 2003, the number of degree-seeking international students from B&R countries^1^ only accounted for 29.71% of the total, but by 2018, the proportion had reached 61.50%. Not only has the number of international students from B&R countries continued to rise, but the proportion is also increasing year by year. This data means that in the future, international students from B&R countries will gradually become the main component of international students in China, with huge development potential.

Traditionally, English-speaking countries are the main destinations of international students. In fact, the existing research has explained the phenomenon that students from developing countries flow to developed Western countries [6,7]. However, as a non-English-speaking, middle-income country, what unique qualities does China possess that attract a significant number of international students? Therefore, the aim of this research is to investigate the influencing factors that drive international students from Belt and Road (B&R) countries to choose China for their studies, as well as to examine the differences in these influencing factors among students at various educational levels. This study intends to provide both theoretical and practical insights into the mobility of international students.

## Literature review

Literature shows that the main reason for choosing to flow to developed countries is that students are extremely concerned about the following aspects of the destination country, including economic conditions [8,9], educational strength [10,11], teaching quality [12] and language similarity and climate [13]. In addition, some students also take the convenience of immigration into consideration [14]. Most scholars have studied the reasons for the flow of international students to developed countries, while there are few articles on the reasons for the flow of international students to developing countries.

In fact, the reasons that influence students’ choice of developed and developing countries are actually different, which may be biased to directly use the literature of research flowing to developed countries to guide developing countries. Addressing this gap, some scholars have studied the influencing factors of international students’ choice to flow to developing countries. The study found that there are five main factors that influence the choice of international students, including scholarship availability [15], family push factor [16], intermediary organization push factor [17], cultural conflicts [18] and lower cost of living and tuition [19], which are not the same as those in developed countries. The main objects of this kind of literature research include developing countries such as India, Malaysia and Thailand. Although these countries attract a significant number of international students, they still lag behind China in terms of economic size, growth, and the strength of higher education. Therefore, it is inappropriate to rely solely on existing literature regarding the choices of international students in developing countries to inform China’s development strategies.

Most of the research on the factors that influence international students to study in China has been done by Chinese scholars. These scholars mainly conducted research on why foreign students choose to study in China from various perspectives, including differences in economic development levels [20,21], cultural acceptance [22,23], the number of Confucius Institutes [24], the quality of education in Chinese universities [25], the status of scholarships [26] and national policy orientation [27]. However, these studies largely consider internal factors related to China and often overlook the international political influences and policy factors, such as the “Belt and Road” initiative, that affect international students’ decisions to study in China. As these factors become increasingly significant in the current developmental context, it is essential to integrate them into the analysis.

In recent years, the evolving landscape of international education has unfolded in three stages [28], influenced by events such as the 9/11 terrorist attacks, the U.S. subprime mortgage crisis, the slowdown of the Chinese economy, the 2016 Brexit referendum, and the 2016 U.S. presidential election. This underscores the importance of the international political climate in shaping students’ choice of destination. Unfortunately, such factors have not been adequately addressed in prior research. Given the current rise in nationalist, populist, and anti-global political sentiments [29,30], it is likely that the determinants of international student mobility will continue to evolve [31]. According to the literature, push-pull theory, human capital theory, and proximity theory dominate the explanations for international student mobility.

### Economic and stability factors: Push-pull theory

In the push-pull theory, the factors that drive students to leave their home countries are called push factors, while the factors that attract students to the destination country are called pull factors. The push-pull theory puts more emphasis on the macro aspects [1,32]. For instance, the ratio of GDP per capita between China and the source country in terms of macroeconomics (div_gdppc), GDP per capita can measure the economic strength of a country, and the ratio of GDP per capita between the two countries can be used to study whether China’s economic development will have an impact on studying in China [33]. The trade volume (trade) between the source country and China indicates the friendly economic and trade relations between the two countries, which can not only reduce the cost of information flow between the two countries to a certain extent, but also have a positive role in promoting the international students [26,34]. At the macro social level, the national stability factor is defined as the difference (diff_stability) between the Political Stability and Absence of Violence/Terrorism index between China and the home countries. The larger the difference, the better the national stability of China compared to the home country. Political stability and the absence of terrorist attacks and violence are the preconditions for the stable development of education in a country. Moreover, a peaceful and stable environment will also encourage local students to study abroad [35]. It is expected that the difference of Political Stability and Absence of Violence/Terrorism indexes has a positive impact on the number of international students.

### Educational factors: Human capital theory

Human capital theory can explain the choice of international students studying abroad from a micro perspective. There is no doubt that when educational resources in their own countries cannot meet their development needs, these students will go to other countries to seek the accumulation of human capital [36–38]. Therefore, in terms of education, mutual recognition of academic degrees between China and the home country (recognition) is the guarantee of educational linkages. It was illustrated by research that the signing of the agreement on mutual recognition facilitated the increase in the number of international students [39], as the agreement on mutual recognition of degrees and diplomas is the effective guarantee of the Chinese degrees recognized by the home country. At the same time, it strengthens the communication about the educational policies between the two countries [40]. The higher education strength of a country can be represented by the number of universities entering the top 500 of the Shanghai Ranking and the number of universities entering the Academic Ranking of World Universities to some extent. The greater the number of top-ranked universities in China, the more attractive they are expected to be to international students [13]. The proportion of international students who receive scholarships (scholarship) is also a factor that cannot be ignored. The scholarship is an important factor considered by international students in the process of studying abroad [15]. It is expected that the larger the proportion of scholarship, the more favorable it will be to promote the growth of the number of international students.

### Sociocultural factor: Proximity theory

The push and pull theory, and human capital theory are more likely to explain the study abroad behaviors driven by tangible factors, but intangible factors such as culture need to be explained by the introduction of proximity theory [41,42]. The geographic distance between the home country and China is also a factor that cannot be ignored. In this paper, the spherical distance between the capital of each home country and Beijing, the capital of China, is incorporated into the model. Generally speaking, the closer the distance between two countries, the lower the transportation cost for international students will be, and it is expected that the number of international students will be affected and increase accordingly [26]. Proximity refers to not only the proximity of space but also the proximity of institutions [43], culture [44] and social relations [45]. These intangible factors also affect international students’ choice of studying abroad. The number of Confucius Institutes in each home country (confucius) is a sociocultural variable. The Confucius Institute is not only a bridge for China to promote the Chinese language and Chinese culture but also an important platform for governmental, public and non-governmental diplomacy [24]. Therefore, it is expected that countries with more Confucius Institutes will have more international students. The similarity between the language of the home country and the Chinese language represents the difficulty of international students learning this new language. The higher the similarity, the faster the international students are likely to master Chinese and communicate better with teachers and classmates [7]. Therefore, it is expected that the higher the language similarity, the greater the number of international students coming to China. The population size of a country (lnpopu) is the basis for the number of international students. A country with a larger population may have a relatively large number of international students. See Table 1 for more details.

**Table 1:** Descriptive statistics.

	Total		B&R		Non-B&R	
	Mean	Std. Dev.	Mean	Std. Dev.	Mean	Std. Dev.
ln(student)	3.96	2.356	4.786	2.315	3.582	2.278
ln(undergraduate)	3.436	2.332	4.234	2.436	3.071	2.189
ln(graduate)	3.024	2.127	3.678	2.124	2.725	2.061
div_gdppc	2.589	4.098	1.827	2.319	2.962	4.685
ln(trade)	20.625	2.756	21.51	2.174	20.19	2.904
ln(distance)	9.012	0.513	8.578	0.377	9.213	0.437
recognition	0.165	0.371	0.252	0.434	0.125	0.33
ranking	24.125	13.131	24.125	13.135	24.125	13.132
scholarship	0.247	0.26	0.18	0.179	0.28	0.287
diff_stability	0.437	1.008	0.180	1.022	0.559	0.978
confucius	1.409	4.983	1.271	2.442	1.472	5.784
language	0.382	0.221	0.439	0.15	0.354	0.243
ln(popu)	15.406	2.221	16.149	1.651	15.048	2.367

In summary, existing research on international student mobility predominantly focuses on developed countries and often lacks a thorough understanding of the unique factors that attract students to developing countries. This may lead to factors and implications that are not applicable to non-Western educational contexts. Additionally, the influence of external political and policy factors on student mobility has not been sufficiently explored in the literature. Therefore, addressing these gaps, our study explores the factors influencing students from B&R countries in choosing to study in a non-English speaking, middle-income country, China. This exploration could provide insights and guidance for formulating higher education strategies that align with the unique position and aspirations of developing countries on the global stage.

## Research design

### Variables and data source

In this context, we choose the number of degree-seeking international students mobility to China as the dependent variable, as degree education more accurately reflects the attractiveness and competitiveness of the host country’s higher education system [46]. For example, in 2018, the number of international students in the United States totaled 1.10 million, of which 0.81 million were degree-seeking international students, accounting for 73.94%, among which graduate students accounted for 34.51% [47]. Therefore, in this research, the dependent variable is defined as the number of degree-seeking international students.

The dependent variable is sourced from the “*Concise Statistics of International Students Studying in China*” compiled by the International Exchange Department of the Ministry of Education of the People’s Republic of China from 2003 to 2018. The dependent variables are divided into the number of international students studying in China from B&R countries and non-B&R countries, and the number of undergraduate and postgraduate students studying in China.

The selection of independent variables in this study is grounded in existing literature, especially the empirical studies by Wei [26], Mazzarol [33], Caruso [35] and West [39], as outlined in the Literature review section. These variables include GDP per capita (div_gdppc), trade volume (trade), the distance between the two countries (distance), mutual recognition of degrees (recognition), the number of top-ranked universities (ranking), international student scholarships (scholarship), political stability (diff_stability), the number of Confucius Institutes (confucius), language similarity (language), and population size (popu), all of which have been identified as influencing international student mobility. These variables were selected based on established theoretical and empirical foundations. Data for these variables primarily come from public databases, including the World Bank, UN Comtrade Database, Shanghai Ranking, and the CEPII Database (see Table 2).

**Table 2:** Variable specifications.

Variable	Definition	Source
ln(student)	The number of degree-seeking international students studying in China	Ministry of Education of China.
Economic factors		
div_gdppc	China’s GDP per capita divided by the home country	World Bank
ln(trade)	Trade volume between China and the home country	UN Comtrade Database
ln(distance)	The spherical distance between China and the home country	CEPII
Education factors		
Recognition	Mutual recognition of academic qualifications between China and the home country	World Bank
Ranking	The number of Chinese universities in the top 500 in the Shanghai Ranking	Shanghai Ranking
Scholarship	Percentage of international students who have received scholarships	Ministry of Education of China.
National stability factors		
diff_stability	China’s Political Stability and Absence of Violence/Terrorism index minus the home country.	World Bank
Sociocultural factors		
Confucius	Number of Confucius Institutes by the home country	Ministry of Education of China.
Language	The degree of similarity between the language of the home country and Chinese	CEPII
ln(popu)	Population of the home country	World Bank

### Method and model

Before conducting regression analysis, the Hausman test was performed to determine the appropriateness of the fixed-effects model over the random-effects model. It was found that the fixed-effects model provided a more suitable framework for our analysis due to its ability to control for unobserved heterogeneity, which could otherwise bias the results. The fixed-effects model has the significant advantage of controlling for invariant characteristics within each cross-sectional unit over time by introducing dummy variables for each individual [48]. This method superiorly addresses the issue of omitted variable bias.

To further refine our analysis, we employed a two-way fixed-effects model, which allows us to control both country-level and year-level specific effects of the panel data. This approach is particularly advantageous as it comprehensively accounts for both spatial and temporal heterogeneity, thus providing more robust estimates compared to the one-way fixed-effects model commonly used in similar studies.

The measurement model was established based on existing research and the theoretical mechanisms discussed previously:


$$ln(studentit)=\alpha_{0}+\beta_{1}div\_gdppc+\beta_{2}ln(trade)+\beta_{3}ln(distance)+\beta_{4}recognition+\;\;\beta_{5}ranking$$

$$+\;\beta_{6}scholarship+\beta_{7}diff\_stability+\beta_{8}confucius+\beta_{9}language+\beta_{10}ln(popu)+\lambda_{i}+\lambda_{t}+\varepsilon_{it}$$

The explained variable ln(student) represents the number of degree-seeking international students who came to China from country i in year t (take the logarithm), α_0_ is a constant term, λ_i_ is a country fixed effect, which includes country-specific unobserved factors that influence the choice of international students, λ_t_ is a year fixed effect, which includes unobserved factors that influence the choice of international students over time, and ε_it_ is an error term. To ensure robustness of our findings, logarithmic transformations were applied to the trade, distance, and population variables. This transformation stabilizes variance and normalizes the distribution of variables, which enhances the interpretability and reliability of the regression results. This methodological refinement offers a clearer insight into the elastic relationships between the predictors and the outcome, compared to simple linear models often used in other studies.

## Results

### Distribution of B&R students’ source countries

**Fig 2:**
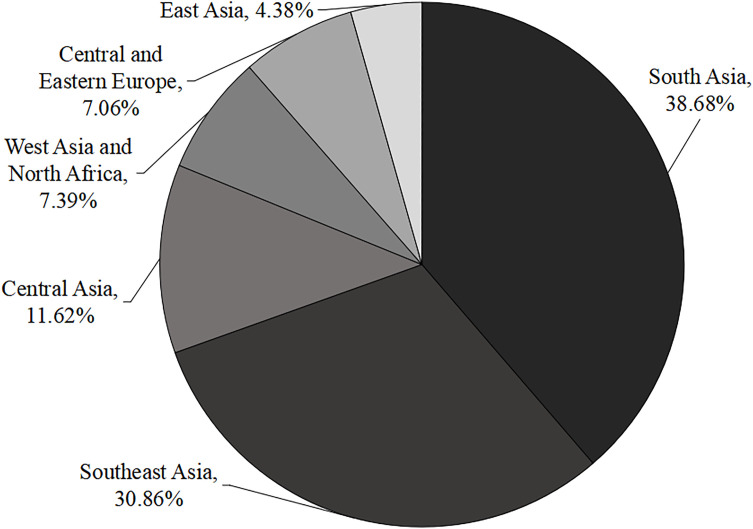
Distribution of B&R students’ source countries. *Source*: Ministry of Education of China. (2018).

The B&R countries include almost all countries in Asia and nearly half of European countries, spanning several regions of the Eurasian continent. Although the B&R countries belong to a whole, there are still great differences within the whole due to the wide distribution range^2^. The regions along the B&R are composed of different countries. These countries are quite different in terms of economic development level, political culture, language customs, and demographic composition [49]. Therefore, the number of international students also varies greatly. As shown in Fig 2, the largest number of international students comes from South Asia and Southeast Asia. South Asia is mainly composed of seven countries with a large population. In 2018, the number of international students totaled 61,401, accounting for 38.68% of the total number, making it the region with the largest number of international students along the B&R. The second is the Southeast Asia region, which is mainly composed of 10 countries. In 2018, the number of international students totaled 48,993, accounting for 30.86% of the total number. It can be seen that the number of students from these two regions accounted for 69.54% of the total number of international students from B&R countries, with a proportion that cannot be underestimated. In contrast, the number of international students from the other four regions is relatively small. In 2018, the number of international students from Central Asia, West Asia, North Africa, Central and Eastern Europe and East Asia was 18,450 (11.62%), 11,725 (7.39%), 11,212 (7.06%) and 6,955 (4.38%) respectively. The number of international students in these 4 regions only accounted for 30.45% of the total. See Table A1 in S1 Appendix for the names of countries in specific regions.

### B&R and non-B&R countries

In terms of economic factors, the GDP ratio between China and the home country has a significant positive influence on the number of international students from B&R countries shown in Table 3 (Coef. = 0.064, *p* < 0.05). The higher the level of China’s per capita GDP, the more international students from B&R countries choose to study in China. This shows that international students choose China because of China’s relatively high level of economic development. Economic development will provide an impetus for educational development [50]. The bilateral trade volume between China and the home country has a significant positive impact on the scale of international students from B&R countries (Coef. = 0.234, *p* < 0.01). The increase in trade indicates that the market of the home country has a greater demand for talents who understand the Chinese market and culture. At this point, international students with Chinese learning experience can turn the benefits of studying abroad into their own advantages by virtue of their comparative advantages in language and other aspects [34,51]. There is no doubt that the increase in trade volume can promote the growth of the number of international students. However, these two economic factors have no significant impact on international students from non-B&R countries. In fact, among non-B&R countries, there are more high-income countries. This shows that China’s economic development may not be an important factor in attracting students from these countries to China. The geographical distance between the two countries has a significant negative impact on the number of international students for both B&R and non-B&R countries, which means that the closer the home country is to China, the more likely it is for international students to choose China. The closer the distance, not only stands for the countries are more likely to have historical and cultural connections [52], but also means that the transportation cost will be relatively low.

**Table 3:** Regression results of international students from B&R countries and non-B&R.

	B&R	Non-B&R
	Ln(student)	Ln(student)
div_gdppc	0.064*	0.021
	(0.025)	(0.013)
ln(trade)	0.234***	−0.052
	(0.052)	(0.034)
ln(distance)	−1.627**	−12.033***
	(0.606)	(2.909)
recognition	−0.171	0.010
	(0.103)	(0.109)
ranking	0.056***	0.061***
	(0.004)	(0.003)
scholarship	0.702***	0.431**
	(0.172)	(0.156)
diff_stability	−0.195***	0.162***
	(0.058)	(0.045)
confucius	0.042***	−0.004
	(0.011)	(0.002)
language	−2.349***	−0.879***
	(0.538)	(0.140)
ln(popu)	0.054	2.689***
	(0.230)	(0.420)
Constant	10.914	44.113***
	(7.996)	(12.650)
Country FE	Yes	Yes
Year FE	Yes	Yes
Observations	923	1,763
R-squared	0.962	0.929

Robust standard errors in parentheses; *** p < 0.001, ** p < 0.01, * p < 0.05; in the model, ln(distance), and language are time-invariant variables. The table presents the coefficients derived from the pooled OLS regression of the three variables.

In terms of educational factors, the mutual recognition of academic qualifications between China and the home country has no significant impact on the B&R countries. This shows that mutual recognition of academic qualifications is not an important factor for international students to consider. For both B&R countries and non-B&R countries, the number of Chinese universities entering Shanghai Ranking top 500 has a significant positive impact. Whenever a university in China enters the top 500, the number of international students from B&R countries will increase by 5.6%. For non-B&R countries, by contrast, the increase will be 6.1%. This shows that the university ranking is indeed an important factor for international students to pay attention to. University rankings can directly reflect the strength of higher education in a country to a certain extent [53]. Scholarships can cover most of the expenses of international students studying and living in China, and therefore have a significant positive effect on international students from B&R countries (Coef. = 0.702, *p* < 0.01). In addition, the Chinese government has also established a special scholarship for the B&R, providing 10,000 government scholarships to international students from B&R countries each year [54]. Therefore, it can be concluded that, compared with non-B&R countries, international students from B&R countries have a greater chance of obtaining scholarships.

In terms of national stability factors, the difference between the Political Stability and Absence of Violence/Terrorism index between China and the home country has a significant negative impact on the scale of international students from B&R countries (Coef. = −0.195, *p* < 0.001). From the perspective of the home country, the higher the political stability of the home country, the lower the value of this factor will be, and the number of international students will increase accordingly. It can be seen that the more stable B&R countries are, the more likely international students will choose to study in China. Domestic stability is a prerequisite for students to study abroad. Meanwhile, the regression results show that the difference between the stability index of China and the home country has a significant positive impact on non-B&R countries (Coef. = 0.162, *p* < 0.001). This shows that the more stable China is, the more international students will choose to come to China. China’s relatively safe environment has laid a solid foundation for attracting international students.

In terms of social and cultural factors, the number of Confucius Institutes has a significant positive impact on the number of international students from B&R countries (Coef. = 0.042, *p* < 0.001). It is well known that Confucius Institutes are platforms for learning Chinese and spreading Chinese Confucian culture. The more Confucius Institutes in the home country, the more opportunities the students have to contact with Chinese Confucian culture, thus having a greater possibility to choose to study in China. The present study aimed to investigate the influence of Confucius Institutes on the number of international students in China. The study found that the influence coefficient of the number of Confucius Institutes is 0.042, indicating that each additional Confucius Institute may lead to a 4.2% increase in the number of international students in China. However, these findings are not entirely consistent with the conclusions reached by Ha and Chen [24], who posited that Confucius Institutes serve as a substitute for studying in China. They contend that international students can obtain Chinese education through Confucius Institutes without the need to physically attend universities in China. This suggests that Confucius Institutes may play a substitutive role in studying in China. Language similarity has a significant negative impact on the scale of international students from both B&R and non-B&R countries. The primary purpose for most international students to study in China is to feel the charm of Chinese while learning Chinese is quite different from other languages of the Indo-European language family [55]. Therefore, the lower the language similarity between the home country and China, the more international students come to China. In addition, the population of the non-B&R country has a positive impact on the number of international students (Coef. = 2.689, *p* < 0.001). Countries with large populations have greater potential for international students to study in China.

### Different degree levels

Among the B&R countries, the ratio of per capita GDP between China and the home country, and the trade volume of the two countries have a significant positive impact on both undergraduates and graduate students, indicating that China’s economic strength and trade have an attractive effect on international students. In terms of educational factors, the mutual recognition of academic qualifications has no significant impact on both undergraduate and graduate students from B&R countries. The number of Chinese universities entering the top 500 in Shanghai Ranking has a significant positive influence on both undergraduates and graduate students. Interestingly, scholarship has a significant positive effect on graduate students from B&R countries, while no significant impact on undergraduates. This is because the setting of scholarships tends to be graduate students. Masters and doctors can create more value through the output of scientific research results. Moreover, attracting more graduate students also represents the high strength of China’s scientific research. In terms of national stability factors, the Political Stability and Absence of Violence/Terrorism index has a significant negative impact on undergraduates from B&R countries. This shows that the more stable home country is, the more international students will choose to come to China. In terms of social and cultural factors, the number of Confucius Institutes has a significant positive impact on both undergraduates and graduate students from B&R countries. Language similarity has a significant negative impact just on the number of undergraduates from B&R countries.

Among non-B&R countries, the ratio of per capita GDP between China and the home country has a significant positive impact on graduate students and no impact on undergraduate students. In contrast, the two countries’ trade volume has a significant negative impact on graduate students. A possible explanation is that the trade products of the two countries may have made international students understand China, which produced a substitution effect and reduced the number of students studying in China. In terms of educational factors, also, the number of Chinese universities entering the top 500 in Shanghai Ranking has a significant positive influence on both undergraduates and graduate students. Although scholarship has no significant positive effect on undergraduates from non-B&R countries, it has a significant positive effect on graduate students. There are many opportunities to obtain scholarships in postgraduate study, so more attention is paid to the scholarships for graduate students. Interestingly, the difference of national stability index has a significant positive effect on both undergraduates and graduate students from non-B&R countries. In terms of social and cultural factors, the number of Confucius Institutes has a significant negative impact on undergraduates from non-B&R countries. Similar to the conclusion of Ha and Chen [24], this shows that the Confucius Institute may have a substitution effect on international students studying in China. On the contrary, the number of Confucius Institutes has no significant effect on the number of graduates. Language similarity has a significant negative impact on the number of undergraduate students from non-B&R countries in Table 4.

**Table 4:** Regression results of undergraduates and graduates from B&R countries and non-B&R.

	B&R		Non-B&R	
	Undergraduate	Graduate	Undergraduate	Graduate
div_gdppc	0.068*	0.199***	0.014	0.024*
	(0.029)	(0.024)	(0.014)	(0.012)
ln(trade)	0.262***	0.116*	−0.021	−0.075*
	(0.054)	(0.056)	(0.042)	(0.032)
ln(distance)	−2.664***	1.478*	−17.774***	−1.651
	(0.734)	(0.600)	(3.354)	(2.794)
recognition	−0.167	0.096	−0.161	0.238
	(0.107)	(0.108)	(0.109)	(0.135)
ranking	0.045***	0.064***	0.053***	0.063***
	(0.005)	(0.005)	(0.004)	(0.003)
scholarship	−0.240	1.403***	−0.235	0.282*
	(0.208)	(0.175)	(0.179)	(0.135)
diff_stability	−0.275***	0.086	0.114*	0.159***
	(0.062)	(0.062)	(0.055)	(0.047)
confucius	0.045***	0.115***	−0.006*	0.004
	(0.013)	(0.014)	(0.002)	(0.003)
language	−3.603***	0.279	−0.839***	5.226
	(0.836)	(0.629)	(0.156)	(20.912)
ln(popu)	0.339	−0.589**	3.463***	1.034**
	(0.266)	(0.213)	(0.488)	(0.399)
Constant	14.812	−5.926	69.313***	0.709
	(9.525)	(7.497)	(14.394)	(12.360)
Country FE	Yes	Yes	Yes	Yes
Year FE	Yes	Yes	Yes	Yes
Observations	923	923	1,763	1,763
R-squared	0.954	0.948	0.902	0.923

Robust standard errors in parentheses; *** p < 0.001, ** p < 0.01, * p < 0.05; in the model, ln(distance), and language are time-invariant variables. The table presents the coefficients derived from the pooled OLS regression of the three variables.

Undergraduate: includes junior college students.

## Discussion

Our study highlights the significant role of economic factors, particularly the trade relationship between the home country and China. This finding aligns with previous studies suggesting that students are often drawn to countries with better economic prospects [6,8,33]. However, our study further underscores the importance of trade relations between the home country and China, which reduces the cost of information flow and creates a stronger pull factor for international students [26,34]. This contribution provides a more detailed understanding compared to prior studies, which primarily focused on broader economic indicators such as GDP.

National stability has emerged as a crucial factor in attracting international students, especially those from B&R countries. This finding aligns with Caruso & De Wit’s argument that a peaceful and stable environment encourages students to study abroad [35]. Further, the analysis in this study demonstrates that both home country and host country stability are important for students from B&R regions, suggesting that B&R students are influenced by the overall political environment, whether stable or unstable.

Interestingly, our analysis revealed that the language similarity in sociocultural factors has a negative impact on international students, which runs counter to some previous studies proposing that language similarity and climate play a significant role in students’ study abroad decisions [13]. This discrepancy might suggest that international students are increasingly seeking exposure to different languages and educational environments. In an increasingly globalized world, many students prioritize the chance to learn new languages, adapt to different educational systems, and gain international perspectives. Moreover, different from the conclusion of Ha & Chen [24], we found that the number of Confucius Institutes is positively related to the number of international students. This may be because Confucius Institutes serve as an entry point for students interested in learning Chinese and immersing themselves in Chinese culture before beginning their formal education in China. Their presence fosters a network of cultural and educational connections, enhancing China’s attractiveness as a destination for international students [56]. The Confucius Institute is a non-profit educational institution run by Sino-foreign cooperation. Although there are some negative comments about Confucius Institutes [57], it is undeniable that Confucius Institutes attract international students.

Regarding educational factors, our study confirms the significance of the number of Chinese universities in the top 500 of the Shanghai Ranking and scholarships for attracting B&R international students, consistent with previous literature highlighting the importance of educational strength and scholarship availability [10,15]. Thanks to the Chinese government’s huge investment in higher education, the number of Chinese universities entering the top 500 of Shanghai Ranking continues to grow. From the “211 Project” and the “985 Project” in the late 20th century to the “Double World-Class” construction in recent years [58], a large amount of capital investment has promoted the rapid growth of the number of high-level papers published [59]. However, mutual recognition of academic qualifications between the two countries did not show a statistically significant effect. This warrant further investigation, perhaps exploring whether other factors such as the reputation of specific institutions or programs of study play a more prominent role.

Existing literature has identified that the factors influencing mobility choices differ between undergraduate and graduate students [60,61]. Therefore, this study also explores the reasons behind the decision of undergraduate and graduate students to study in China. We find that graduate students are more likely to be attracted by China’s economic development potential and scholarships. This is because they may perceive academic research opportunities, participation in research projects, or the possibility of receiving scholarships in China as ways to enhance their personal competitiveness and career development [62]. Additionally, scholarships can significantly alleviate their financial burden, especially at the graduate level, where economic pressures are often greater than those faced by undergraduates. In contrast, undergraduates may place more importance on socio-cultural factors such as national stability and cultural exchange opportunities. For many undergraduates, the decision to study abroad is not only motivated by academic knowledge but also by the desire to experience different cultures and lifestyles [63]. China’s political and social stability makes the study abroad experience safer and smoother, while abundant cultural exchange opportunities offer undergraduates a comprehensive international education experience.

As for the differences between students from B&R countries and non-B&R countries, we found that students from B&R countries are more influenced by China’s economic factors and trade relations, reflecting the economic integration promoted by the B&R Initiative. These students may view studying in China as a gateway to understanding the Chinese market [64], whereas students from non-B&R countries are less influenced by these factors and tend to focus more on educational reputation and scholarships. Additionally, international students from B&R countries are more sensitive to cultural factors. This is due to the historical and geographical connections between B&R countries and China, which often make students from these regions more adaptable to Chinese culture [65]. The Confucius Institutes play a key role in this regard, as they provide organized Chinese language education and cultural immersion opportunities, helping students from B&R countries feel more supported and connected to China [24].

The findings of our study offer several key implications for countries aiming to attract international students.

First, national-level policies play a crucial role. Like China’s Belt and Road Initiative, other nations can benefit from strategic, top-down policies that focus on international student recruitment. By aligning educational strategies with broader national goals, such as economic development and cultural exchange, countries can create a competitive edge in the global higher education market.

Second, policies to attract international students should be tailored to different student characteristics. For graduate students, strategies should focus on enhancing academic and research opportunities, including offering scholarships and fostering strong university reputations. In contrast, attracting undergraduate students may require cultural strategies, such as promoting language programs and fostering social integration. Soft power can play a significant role in appealing to younger students looking for both educational and cultural experiences [66].

Finally, economic development and national stability are fundamental and important factors in international student mobility. Countries aiming to attract international students should prioritize economic growth [67] and maintain a stable political environment. Policymakers should ensure that the economic conditions are conducive to educational opportunities and that stability is promoted through both domestic and foreign policies. In this context, fostering an environment of security and growth can be key to attracting international students and ensuring sustained mobility.

In summary, attracting international students requires a multifaceted approach that includes national policies, tailored strategies for different student groups, and a focus on economic and political stability. By investing in both academic excellence and cultural diplomacy, countries can improve their standing in the global education landscape. As the B&R initiative advances, it is likely that China will exert a significant influence on the pattern of overseas study in Asia and possibly beyond [68]. Our findings, therefore, could provide insights for policy-makers and higher education institutions on how to more effectively attract and serve international students in this changing landscape.

## Conclusion

The contribution of this study lies in the integration of push-pull theory, human capital theory, and proximity theory to develop a comprehensive analytical framework that explains the multi-dimensional factors influencing international students’ decisions to study in China, a non-English-speaking, middle-income country. Additionally, by incorporating the perspective of the Belt and Road Initiative, this study distinguishes between B&R countries and non-B&R countries, as well as between different degree levels (undergraduate and graduate). This categorization provides more detailed and targeted insights, which can contribute to the development of more precise policies for attracting international students.

In the analysis, it is mainly found that economic factors are basic attraction factors for international students, while the trade exchanges between the home countries and China can significantly promote the increase in the number of international students. This study also finds that the national stability factor has a strong effect on international students from B&R countries. The stability of the home country and China are both important factors. In addition, the language similarity in social and cultural factors has a negative impact on international students. What international students hope for going abroad is to have more exposure to different languages and educational environments, which fully demonstrates the truth that “distance produce attraction”. The number of Confucius Institutes has a positive impact on the number of international students. For every additional Confucius Institute established in a country along the B&R, the number of international students in China may increase by 4.2%. Among the educational factors, the number of Chinese universities entering the top 500 of Shanghai Ranking, and scholarships have a significant positive impact on B&R international students. In contrast, the mutual recognition of academic qualifications between the two countries is not significant.

From the perspective of B&R countries and non-B&R countries, economic and social and cultural factors have a higher influence on international students from B&R countries. Among the educational factors, the scholarship and Chinese university ranking have a significant influence on both B&R countries and non-B&R countries. National stability factors have different impacts on B&R countries and non-B&R countries. For students from B&R countries, National Stability Index has a significant negative impact, while for students from non-B&R countries, it has a significant positive impact. Next, proceed from the perspective of undergraduate and graduate students. Undergraduates usually pay more attention to national stability and sociocultural factors, while graduate students pay more attention to China’s economic factors and educational strength.

## Limitation

While this study contributes to the literature on international education, it is not without limitations. About the data, the Ministry of Education of China has only released data on international students up to 2018. The onset of the global pandemic in late 2019 disrupted the publication of subsequent data, and even with the pandemic’s end in 2023, no updated data have been made available. Consequently, this study is confined to analyzing data available prior to 2018. The pandemic has profoundly affected global higher education and international student mobility, likely complicating the factors that influence the decision to study in China. Future research could benefit from conducting surveys and interviews with international students at various Chinese universities to delve deeper into their motivations for choosing China as their study destination.

## Supporting information

S1 Dataset Data about international students mobility to China. (XLSX)

S1 Appendix Brief profile of B&R countries. (DOCX)
