# Supplementary material for: The determinants of international students studying in China: An empirical research based on the “Belt and Road” perspective
Source: PLoS One. 2025 Aug 26;20(8):e0329787. doi: 10.1371/journal.pone.0329787 (PMC12380349; doi:10.1371/journal.pone.0329787)
Supplement: S1 Appendix — (DOCX) [file pone.0329787.s002.docx]

# Appendix

**1**：“Belt and Road” is the abbreviation of “Silk Road Economic Belt” and “21st-Century Maritime Silk Road”. In September 2013, Chinese President Xi Jinping proposed the “Belt and Road” Initiative. To borrow the historical symbols of the ancient Silk Road, the “Belt and Road” Initiative holds high the banner of peace and development, actively develops economic partnership with countries along the Belt and Road, and jointly builds a community of shared interests, shared future and shared responsibilities with political mutual trust, economic integration and cultural inclusiveness. In terms of education, the “Belt and Road” initiative emphasizes the coordinated and common development of countries along the route. The educational program of the “Belt and Road” initiative aims to bring unprecedented academic exchanges between China and countries along the New Silk Road and Maritime Silk Road, and at the same time to cultivate a group of “Chinese known and friendly” international talent groups.

Based on historical and geographical connections, there are 65 countries that have been included in the B&R as countries that are along the Belt and Road (hereinafter called B&R countries). The list of specific countries is in Table A1.

**2**: Based on geographic proximity, economic development level and related research expression habits, the West Asia and North Africa B&R countries will be collectively referred to as West Asia and North Africa, and the European B&R countries will be collectively referred to as Central and Eastern Europe in the following analysis.

**Table A1** 65 B&R countries

| Southeast Asia (10) | Indonesia, Thailand, Malaysia, Vietnam, Singapore, Philippines, Myanmar, Cambodia, Laos, Brunei |
| --- | --- |
| East Asia (1) | Mongolia |
| South Asia (7) | India, Pakistan, Bangladesh, Bhutan, Sri Lanka, Nepal, Maldives |
| Central Asia (5) | Kazakhstan, Uzbekistan, Turkmenistan, Kyrgyzstan, Tajikistan |
| West Asia and North Africa (21) | Saudi Arabia, Oman, UAE, Georgia, Azerbaijan, Iran, Turkey, Israel, Kuwait, Iraq, Qatar, Jordan, Lebanon, Bahrain, Yemen, Syria, Armenia, Palestine, Afghanistan, Cyprus, Egypt |
| Central and Eastern Europe (21) | Russia, Ukraine, Belarus, Moldova, Latvia, Lithuania, Estonia, Poland, Czech Republic, Slovakia, Hungary, Romania, Bulgaria, Slovenia, Croatia, Albania, Greece, Serbia, Macedonia, Bosnia and Herzegovina, Montenegro |

Due to lack of data on language similarity or the gross enrollment rate of higher education, these 7 B&R countries have not entered the model, such as Myanmar, Mongolia, Maldives, Palestine, Serbia, Bosnia and Herzegovina, and Montenegro.
